# Cannabinoids in Motor Control: From Receptor Distribution to Motor Disorders

**DOI:** 10.3390/biomedicines14040844

**Published:** 2026-04-08

**Authors:** Dan Faganeli, Metoda Lipnik-Stangelj

**Affiliations:** Institute of Pharmacology and Experimental Toxicology, Faculty of Medicine, University of Ljubljana, Korytkova 2, 1000 Ljubljana, Slovenia

**Keywords:** cannabinoids, cannabinoid 1 receptor, cannabinoid 2 receptor, motor control, basal ganglia, muscle tone, motor disorders, movement disorders, spasticity

## Abstract

Cannabinoid receptors occupy strategic control nodes within motor circuitry, making them potential targets for modulating different motor manifestations. They are positioned both within basal ganglia circuits that regulate movement and within spinal circuits that control skeletal muscle tone. Consequently, cannabinoids have been studied across diverse motor disorders, most notably in movement disorders and tone disorders, particularly those resulting in spasticity. Because motor control spans multiple anatomically and functionally distinct levels, relating cannabinoid signaling to effects on motor function is not straightforward. Limited understanding of cannabinoid receptor distribution has led to cannabinoids being tested even in disorders where receptor localization would predict little or no benefit. Mapping receptor distribution within individual motor circuits and integrating them with their pharmacological effects can help anticipate how cannabinoid signaling shapes motor output. Combined with characteristic motor manifestations, one can identify motor disorders in which cannabinoids may have therapeutic value. In this review, we integrate existing evidence to place cannabinoid receptors within key motor pathways, ranging from basal ganglia circuits controlling movement to peripheral mechanisms governing muscle tone. We consider both cannabinoid 1 receptor (CB_1_R) and cannabinoid 2 receptor (CB_2_R), with CB_2_R gaining attention only recently for its potential relevance within the central nervous system. Building on this framework, we infer how cannabinoids acting at these sites may modulate motor control, and consequently, influence motor manifestations across major motor disorders. Finally, we examine how these distribution-based expectations align with available clinical observations.

## 1. Introduction

The endocannabinoid system is recognized as a major modulatory signaling network that shapes synaptic and cellular functions across the body. It is defined by cannabinoid receptors and their endogenous ligands, the endocannabinoids. These receptors are strategically positioned, most commonly at neuronal synapses, where they support activity-dependent, local control of neurotransmission. Following excitatory stimulation, endocannabinoids are typically synthesized in the postsynaptic membrane and released into the synaptic cleft, acting retrogradely on presynaptic cannabinoid receptors to modulate additional neurotransmitter release. The principal endocannabinoids, 2-arachidonoylglycerol (2-AG) and anandamide (AEA), are produced de novo from membrane phospholipids [1]. In this respect, they resemble prostaglandins, which are likewise generated de novo from membrane precursors, and once released, modulate signaling in neighboring cells in a paracrine manner.

As the expression sites and distribution of cannabinoid receptors have been mapped in greater detail, the role of the endocannabinoid system has become increasingly clear. Cannabinoid receptors are well-characterized in peripheral tissues, where they modulate, among other functions, the enteric nervous system and reduce gastrointestinal motility [2]. A substantial proportion is also expressed within the central nervous system (CNS). In general, knowledge of the anatomical distribution of cannabinoid receptors allows inferences about the functions that cannabinoids are likely to modulate. In several nuclei and pathways of the CNS, receptor distribution is already well-established, enabling relatively straightforward explanations on effects such as appetite via hypothalamic receptors or impaired memory via hippocampal receptors [3,4].

In contrast, relating cannabinoid signaling to motor function is less direct. Motor control is distributed across multiple anatomically and functionally distinct levels, extending from cortical and basal ganglia circuits involved in movement selection and planning to spinal networks and muscle spindles governing skeletal muscle tone. Moreover, modulation of even adjacent subregions within the same anatomical area can markedly influence motor outcomes. The difference between a hyperkinetic and a hypokinetic disorder, for example, may depend only whether modulation occurs at the primary or secondary neuron within same basal ganglia loop. Such organization provides a rationale for why the same cannabinoids have been explored clinically across motor disorders with apparently opposing motor manifestations, such as those in Parkinson’s disease and in Huntington’s disease. Reviews that would attempt to integrate cannabinoid receptor distribution across motor pathways are currently lacking. Such a framework would help explain how cannabinoids influence different motor outcomes. Although numerous reviews address cannabinoid effects in motor disorders, they typically adopt a disorder-specific approach and interpret mechanisms within that single clinical context [5,6,7]. As a result, the literature on cannabinoid involvement in system-level motor control remains fragmented. Integrative publications that do exist are largely restricted to basal ganglia circuitry and are now more than a decade old [8,9,10]. Such studies also provide only a limited coverage of CB_2_R. Only recently has the potential relevance of CB_2_R gained attention due to reports suggesting their broader distribution and inducible expression within the CNS [11]. An incomplete understanding of cannabinoid receptor distribution has led to cannabinoids being tested clinically across a broad range of motor disorders, even in cases where receptor localization would allow one to anticipate that any therapeutic effect will be minimal or absent.

This review will therefore try to integrate existing evidence to place cannabinoid receptors within all key motor pathways, ranging from basal ganglia circuits controlling movement to peripheral mechanisms governing muscle tone. Building on this framework, we will infer how cannabinoids acting at these sites may modulate motor control, and consequently, influence motor manifestations across major motor disorders.

## 2. Motor Control and Dysregulation

The motor system is a broad term used to describe all the central and peripheral structures that support motor behavior [12]. One way to classify their involvement in motor control is phenomenologically, meaning by the manifestations one sees at the patient’s bedside, especially in the case of dysregulation (see Box 1). Most broadly, the motor system is involved in controlling two principal categories: movement and muscle-tone. This distinction reflects whether the primary difference is expressed in the quantity or quality of movement, or in the resistance of a muscle to passive stretch. In the case of movement dysregulation, it can either be hypokinetic, where it is reduced or slowed, or hyperkinetic, where it is characterized by excessive, involuntary, or inappropriate moves.

Box 1 **Phenomenological classification of motor dysregulation with related manifestations.**

**Phenomenological classification of motor dysregulation with related clinical manifestations**
 Movement disorders:Hypokinetic: akinesia, bradykinesia, reduced amplitude (Example: Parkinson’s disease)Hyperkinetic: chorea, dystonia, myoclonus, tics and other dyskinesias (Example: Huntington’s disease)Tone abnormalities:Increased tone: spasticity, rigidity, paratonia, tonic dystonia (Example: multiple sclerosis, cerebral palsy, stroke)Decreased tone: flaccidity, hypotonia (Example: spinal muscular atrophy, peripheral neuronal lesions, myopathies)Accompanied by strength disorders: paresis, paralysisCoordination and gait disorders:
Ataxia, dysmetria

Muscle-tone control, in contrast, is reflected in the degree of baseline muscle resistance to passive movement. If it is pathologically increased, it manifests as spasticity or rigidity, or if decreased, it is seen as flaccidity or hypotonia. Tone abnormalities frequently coexist with strength deficits, since both functions depend on the integrity of upper motor neurons, lower motor neurons, and the peripheral musculature.

In addition to movement and muscle tone, the broader motor system also controls coordination, which relies predominantly on cerebellar processing. Disruption of these circuits can lead to manifestations such as ataxia, dysmetria, and other impairments of movement timing and accuracy. In contrast, the primary mechanistic scaffold for the motor manifestations addressed is provided by basal ganglia and spinal loops. Cerebellar contributions are not negligible, but they are likely to be more important for movement refinement than for a first-order explanation of the phenomena discussed here. Moreover, the integration of cerebellar circuits into motor control is highly complex and would warrant a separate dedicated analysis beyond the scope of the present review. Accordingly, although coordination may be considered part of motor control in a broader sense, the present review focuses on motor control in the narrower, predominantly basal ganglia- and spinal-related sense.

### 2.1. Regulation of Movement

**Figure 1:**
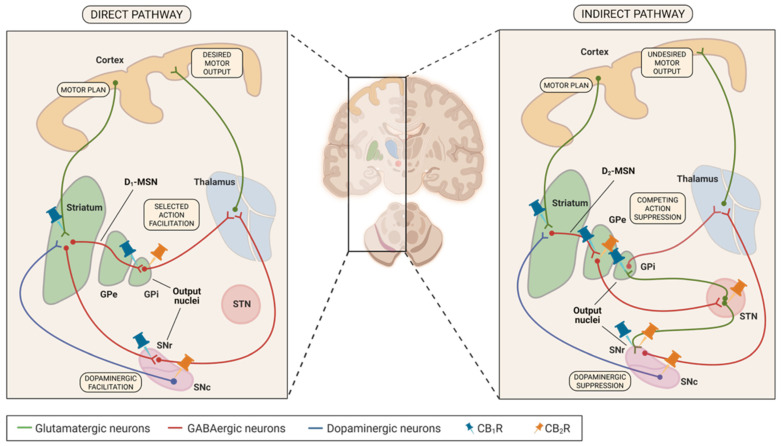
Basal ganglia circuits involved in movement with CB receptor distribution *. In the direct pathway, cortical glutamatergic input excites D_1_ receptor-expressing MSNs (D_1_-MSNs) in the striatum. These MSNs are GABAergic and project directly to the internal segment of the globus pallidus (GPi) and substantia nigra pars reticulata (SNr), which themselves are GABAergic output nuclei. At rest, GPi/SNr neurons fire tonically and inhibit the motor thalamus. When the direct-pathway MSNs are activated, their GABAergic inhibition of GPi/SNr reduces this tonic inhibitory outflow. The thalamus is thereby disinhibited, increases its glutamatergic drive to motor cortex, and voluntary movement is facilitated in the pyramidal tract. In the indirect pathway, cortical glutamatergic input excites a different population of GABAergic MSNs that primarily express D_2_ receptors (D_2_-MSNs). These project first to the external segment of the globus pallidus (GPe), which is also GABAergic and normally inhibits the subthalamic nucleus (STN). Activation of D_2_-MSNs inhibits GPe, relieving its GABAergic brake on the STN. The disinhibited glutamatergic STN then increases its excitatory drive onto GPi/SNr. This boosts GABAergic inhibition from GPi/SNr to the thalamus, reducing thalamocortical glutamatergic output and thereby suppressing movement. Both direct and indirect tracts are modulated with dopaminergic neurons projecting from the substantia nigra pars compacta (SNc) to the striatum. Dopamine in the striatum acts on MSNs, which besides glutamatergic receptors also express dopaminergic receptors. Indirect-pathway D_1_-MSNs express stimulatory D_1_ receptors, causing dopamine from SNc to enhance their activity. Indirect pathway D_2_-MSNs express inhibitory D_2_ receptors, causing dopamine to inhibit them. Thus, dopamine simultaneously strengthens the pro-kinetic direct pathway and weakens the anti-kinetic indirect pathway, biasing this system toward movement. Figure was created in BioRender. Faganeli, D. (2026) https://BioRender.com/i0rk94v, accessed on 30 March 2026. * Only sites with robust CB receptor expression are indicated.

Gating, selection, and scaling of movement action happens in the basal ganglia. The motor cortex then constructs and sends out the detailed movement commands. In the basal ganglia, movement is regulated by two intertwined circuits, the direct and indirect pathways. Both begin in the striatum, which receives glutamatergic projections from the motor cortex, particularly when a desired move is to be initiated. The striatum contains GABAergic medium spiny neurons (MSNs) that project to the output nuclei, globus pallidus internus (GPi), and substantia nigra pars reticulata (SNr). These projections regulate the inhibitory output to the thalamus, thereby controlling how much thalamic excitation is returned to motor cortex and how a specific movement is expressed (see Figure 1).

### 2.2. Muscle Tone Control

When a signal for movement passes from the motor cortex, corticospinal neurons provide the precise, fractionated command to peripheral neurons. This causes coordinated muscle contraction, resulting in movement. However, even without such signals for a voluntary move, in an awake, healthy person, most postural muscles and many limb muscles still have some baseline action potential firing, seen as skeletal muscle tone.

**Figure 2:**
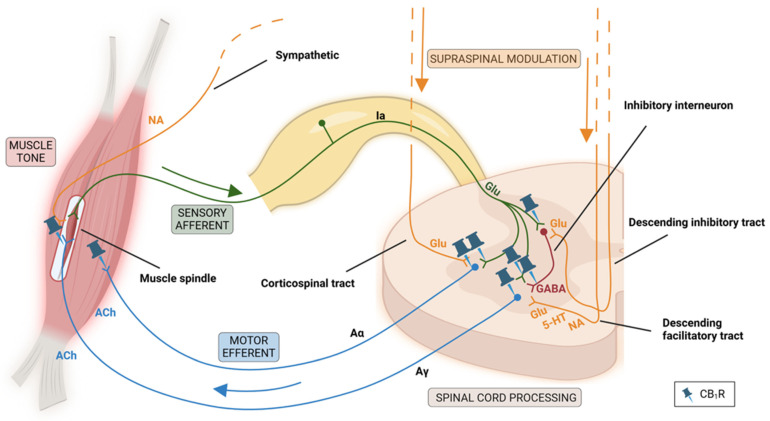
Spinal circuits involved in muscle tone with CB receptor distribution *. At the neural level, skeletal muscle tone is primarily implemented by spinal stretch-reflex circuits. When muscle is lengthened, sensory axons from muscle spindles, such as Ia, send signals to the spinal cord. In the ventral horn, they make an excitatory connection onto the extrafusal muscle’s α-motoneurons, largely through glutamatergic (glu) transmission. In this way, they increase α firing and produce a restoring contraction. Ia afferents simultaneously activate interneurons that presynaptically inhibit Ia terminals, thereby reducing stretch reflex gain in a negative feedback manner. The spindle itself is actively tuned by γ-motoneurons (Aγ) that regulate spindle tension, thereby setting the gain of the stretch reflex. Supraspinal systems set the operating point by influencing motoneuron excitability and setting the reflex gain. Main facilitatory descending influences are pontoreticulospinal and vestibulospinal pathways, central to posture and balance. They provide a tonic excitatory drive to extensor-related spinal circuits and simultaneously raise their stretch-reflex gain to make antigravity muscle tone sustainable. Overlaying this, descending noradrenergic (NA) and serotonergic (5-HT) projections can further facilitate the same spinal circuits by acting on excitatory receptors in the ventral horn, both on motoneurons and interneurons. In contrast, bulboreticulospinal neurons provide inhibitory influence on spinal motor circuits, largely by engaging spinal inhibitory interneurons and by the presynaptic inhibition of Ia afferent terminals, so that stretch inputs do not produce excessive reflex contraction. Normal muscle tone reflects the dynamic balance between these facilitatory and inhibitory tendencies. Finally, muscle tone can also be influenced peripherally at the muscle via sympathetic activity. Skeletal muscle expresses β2-adrenergic receptors involved in metabolic regulation, which increases contractile responsiveness. Figure was created in BioRender. Faganeli, D. (2026) https://BioRender.com/wfppnwi, accessed on 25 February 2026. * Only sites with robust CB receptor expression are indicated.

Such muscle tone is produced by two coupled processes, continuous stretch-reflex micro adjustments and a tonic descending drive that sets spinal excitability. Continuous muscle micro-stretches and sway activate muscle spindle afferents and thus engage the spinal stretch reflex causing constant muscle micro adjustments. In an awake person, postural muscles additionally receive tonic baseline excitation from extrapyramidal tracts (reticulo-/vestibulospinal tracts), providing drive to lower motor neurons (LMNs) innervating muscles involved in posture [13]. Additionally, monoaminergic descending pathways also tune the gain of the stretch reflexes, so that a given degree of stretch produces an appropriately sized corrective response. This way, the muscle tone is constantly being tuned for different situations. For example, it decreases while sleeping, in coma, etc. and increases when standing upright, with alertness and anxiety (see Figure 2).

## 3. Cannabinoids in Motor Control

To be directly involved in motor control, cannabinoids must first modulate neuronal excitation. With the anatomical distribution of cannabinoid receptors and their direct effect on neuronal excitability, one can explain how cannabinoids modulate motor output. The general effects of cannabinoid receptors on metabolism, proliferation, and general homeostasis are important for chronic processes such as synaptic plasticity and regeneration [14]. However, these effects are of lesser importance in the case of both movement execution and tone modulation.

Two main cannabinoid receptors have been characterized, CB_1_R and CB_2_R, both G protein-coupled receptors (GPCRs) that couple primarily to G_i/o_ proteins. In both cases, their activation inhibits adenylyl cyclase, suppresses voltage-gated Ca^2+^ channels, activates several inwardly rectifying potassium channels, and ultimately reduces neuronal excitability [1].

CB_1_R is abundant in different circuits involved in motor control. Typically, it is heavily concentrated presynaptically on axon terminals, where it mostly acts as a fast synaptic and excitability regulator. Since CB_1_R is G_i/o_-coupled, it rapidly suppresses transmitter release by inhibiting Ca^2+^ channels and modulating vesicle machinery; this way, in motor control, CB_1_R is wired into different circuits as a classic synaptic knob [15].

On the other hand, CB_2_R is expressed predominantly in immune and hematopoietic cells and was long thought to be absent from a healthy brain [16]. However, newer studies suggest that CB_2_R is also present in various parts of the CNS including the striatum, pallidum, substantia nigra, and basal thalamus [17]. Still, compared to CB_1_R, the presence of CB_2_R is much lower and more condition-dependent, and a lot, if not most of the signal, is in the glia and microglia. CB_2_R in the CNS is expressed in orders of magnitude lower compared to peripheral immune tissues [18]. The expression on neurons themselves is even more negligible, frequently causing the CB_2_R signal from the glia to overshadow the neuronal signal [19]. CB_2_R in the CNS is consequently mostly studied for its effect on metabolic and proliferative functions and not for its effect on direct neuronal excitability [20,21]. Nevertheless, there are studies that show that CB_2_R is also at least partially directly involved in neuronal excitation, most commonly of dopaminergic pathways [18,22,23].

Beyond CB1 and CB2, several other GPCRs have been proposed to participate in cannabinoid-related signaling, most notably GPR55, GPR18, and GPR119. These receptors are considered putative cannabinoid-related GPCRs rather than classical cannabinoid receptors because their pharmacology and the physiological relevance of their proposed endogenous ligands remain less firmly established [24]. Of these, only GPR55 has shown meaningful motor-related evidence including expression in movement-related nuclei and modest preclinical motor effects in rodent studies [25]. However, these findings remain preclinical and far less robust than the extensive evidence for CB_1_R or even CB_2_R. Accordingly, these receptors are unlikely to be of major relevance to direct motor control.

## 4. Distribution of Cannabinoid Receptors in Motor Circuits

### 4.1. Involvement of CB Receptors in Basal Ganglia—Direct Pathway

In the direct pathway, CB_1_Rs localize predominantly to presynaptic terminals on both corticostriatal glutamatergic inputs and striatal GABAergic outputs (See Figure 1). Consistent with the predominant CNS pattern, CB_1_Rs within basal ganglia circuits are largely presynaptic. In the case of corticostriatal projections, this results in CB_1_R labelling within the striatum, which is in line with both functional [26] and immunohistochemical evidence [27]. Likewise, CB_1_R is also present on D_1_-MSN terminals in the basal ganglia’s output nuclei, namely the GPi. First, studies to suggest the latter were conducted using autoradiography [28] and immunohistochemistry [29], which showed binding of the CB_1_R marker in the globus pallidus internus (GPi) and pars reticulata of substantia nigra (SNr). After selective destruction of the striatum containing somata of D_1_-MSN, binding of the CB_1_R marker in globus pallidus disappeared. A similar conclusion can be obtained from gene expression studies [30,31,32,33] and electron microscopy [30,34]. While most CB_1_Rs are observed in terminal ends located in the output nuclei, a subset of D_1_-MSNs also gives rise to local axon collaterals ending in the striatum, where the CB_1_R signal is also present [30,34] (Figure 1). Consequently, CB_1_Rs can suppress GABAergic transmission to the output nuclei, as demonstrated with electrophysiological recordings [35].

Nigrostriatal dopaminergic neurons, which provide dopamine that modulates D_1_- and D_2_-MSNs in the striatum, show a different pattern. In general, dopaminergic neurons, including nigrostriatal neurons, do not express CB_1_Rs [36]. Studies using tyrosine hydroxylase labelling identify dopaminergic neuronal somata, whereas the absence of CB_1_R transcripts and CB_1_R immunostaining argues against CB_1_R expression by these neurons [28,29]. Fluorescence in situ hybridization (FISH) provides additional support for this conclusion [37].

Besides CB_1_Rs, CB_2_Rs have also been described within direct-pathway circuitry. Most prominently, they are localized on pallidothalamic GABAergic output neurons and nigrostriatal dopaminergic neurons. Unlike CB_1_Rs, which localize predominantly to presynaptic terminals, CB_2_Rs appear mainly somatodendritic. In macaques, hybridization-based approaches report CB_2_R labelling specifically on the somatodendritic membranes of pallidothalamic neurons [38]. CB_2_Rs have also been reported on midbrain dopaminergic pathways. While CB_1_Rs are generally not detected on the somatodendritic membranes of these neurons, nigrostriatal dopaminergic neurons represent a second major CB_2_R source within direct-pathway-related circuitry [6,23]. In mice, immunofluorescence studies localize CB_2_R predominantly to the somatodendritic compartment of nigrostriatal neurons in SNc [39], while electrophysiological recordings support functional CB_2_R signaling in these neurons [39,40,41]. Through this somatodendritic localization, CB_2_Rs primarily affect dopaminergic output to both D_1_- and D_2_-MSNs, rather than acting primarily via the presynaptic control of MSN terminals, as is typical for CB_1_R. Certain reviews also tend to report the presence of CB_2_R on nigrostriatal terminals in the striatum, however, direct, definitive localization to DA terminals is less consistent and often inferred from functional studies [42] rather than clean anatomical proof. Additionally, some evidence suggests low CB_2_R expression in striatal projection neurons, which can increase after metabolic or excitotoxic insults [43]. These data imply that D_1_-MSNs and D_2_-MSNs possess some somatodendritic CB_2_Rs, however, direct modulation of MSNs’ GABA release is still attributed mainly to CB_1_Rs from their terminal ends.

### 4.2. Involvement of CB Receptors in Basal Ganglia—Indirect Pathway

Since both direct and indirect pathways share most of their anatomical locations, cannabinoid receptor expression in both is mostly similar. Receptor expression appears primarily region-, rather than pathway-, dependent. Accordingly, receptor profiles for shared components, including in nigrostriatal dopaminergic projections, are treated as the same here and are not repeated. The main differences arise at pathway-specific elements, particularly in the subthalamic nucleus (STN), which participates only in the indirect pathway, and D_2_-MSNs, which project to the external globus pallidus (GPe) rather than the GPi (See Figure 1).

Even though D_2_-MSNs express distinct dopamine receptors, they seem to resemble their D_1_ counterparts in terms of CB_1_R expression, consistent with their similar anatomical organization. As in the direct pathway, the main CB_1_R function in the indirect pathway is the presynaptic inhibition of D_2_-MSN axonal terminals, which unlike the direct pathway, terminate in the external globus pallidus (GPe). The GPe displays some of the highest CB_1_R binding in the brain. Lesions of striatum sharply reduce the CB_1_R signal in GPe, and slice recordings show that CB_1_R agonists depress GABA release from striatopallidal terminals, with effects blocked by CB_1_R antagonists [30,44]. CB_1_Rs are also present on terminals of D_2_-MSN collaterals within the striatum where it contributes to endocannabinoid-dependent short-term plasticity [34,35] (Figure 2). Some CB_1_Rs have also been reported on somatodendritic parts of D_2_-MSNs located in the striatum, as demonstrated with mRNA and immunostaining [31,32,33], however, less compared to their axonal counterparts.

STN neurons form glutamatergic projections to the GPi and SNr and express CB_1_Rs on their axon terminals, consistent with the predominantly presynaptic localization of CB_1_Rs. Studies show that the stimulation of STN projection neurons evokes excitatory postsynaptic currents (EPSCs) in both SN [45,46] and GPi [47]. In those studies, CB_1_R agonists reduced EPSC while CB_1_R antagonists restored them, without a change in postsynaptic sensitivity to glutamate, indicating that CB_1_Rs are located on axon terminals.

Within the indirect pathway, CB_2_Rs show similar regional distribution patterns to that of its direct-pathway counterparts. Beyond the locations shared with the direct pathway, CB_2_Rs are also expressed at several nodes of the indirect pathway. Pallidal neurons, projecting from GPe to STN, express CB_2_Rs on their somatodendritic ends located in GPe [38]. This parallels direct-pathway CB_2_R expression in the somatodendritic compartment of pallidal neurons in the GPi, again supporting the view that CB_2_R expression is region-, rather than pathway-dependent. In contrast, neurons projecting from STN provide a clear example of CB_2_R expression in both somatodendritic and their axonal compartments in SNr. Studies have shown CB_2_R mRNA transcripts and CB_2_R immunostaining on slices and primary STN cultures. Electron-microscopy studies combined with studies of cannabinoids’ effects on presynaptic glutamatergic release additionally demonstrate CB_2_R presence on their axon terminals in SNr [48]. Consistent with regional analogies, similar CB_2_R presence should exist at STN terminals in GPi, as the other site of the projection, though this remains yet to be documented.

### 4.3. Involvement of CB Receptors in Spinal Cord Reflex Circuitry

In circuits involved in spinal cord reflexes governing muscle tone, CB_1_Rs are demonstrably present at several key strategic nodes. Primarily, they are expressed on presynaptic membranes of primary afferent neurons, in defined dorsal horn interneuron populations, in ventral horn motoneuron somatodendritic compartments, and at the neuromuscular junction (NMJ) on the presynaptic motor terminal (see Figure 2).

In primary afferent neurons, CB_1_Rs are present both in sensory neuron somata as well as their terminals. Molecular and immunohistochemical approaches demonstrated CB_1_R mRNA and CB_1_R protein presence already in perikarya of the dorsal root ganglion (DRG) [49]. Nevertheless, a substantial fraction of spinal cannabinoid receptors resides on primary afferent terminals. In a classic autoradiographic study, unilateral dorsal rhizotomy reduced spinal cannabinoid radioligand binding on the injured side by roughly half, indicating that a substantial fraction of CB_1_Rs in the dorsal horn is associated with primary afferent terminals [50]. Complementary immunocytochemical work reported CB_1_R immunolabeling in heterogeneous DRG neurons, more specifically on their axons in Lissauer’s tract [51]. These studies provide strong evidence for presynaptic CB_1_Rs on various primary afferent inputs to the spinal cord, but they do not, on their own, identify CB_1_R specifically on the terminals of Ia muscle spindle afferents.

Within intrinsic spinal circuitry, CB_1_Rs have been localized to defined interneuron populations, most clearly in the dorsal horn. Most work has characterized CB_1_Rs in the context of sensory processing and gating of pain. Nevertheless, similar interneuron-mediated mechanisms that gate nociceptive inputs also shape the afferent drive that feeds into spinal reflex circuits, making this literature also relevant from a motor-control standpoint. Immunocytochemical mapping in the rat spinal cord suggests that a substantial portion of CB_1_R immunoreactivity arises from intrinsic spinal interneuron networks [52]. Double-labelling additionally indicates that CB_1_R-positive dorsal horn interneurons are inhibitory including GABAergic or NO synthase-positive [51]. What remains notably less resolved, however, is whether CB_1_Rs can be assigned to the specific interneuron classes commonly invoked in muscle tone control such as the interneurons mediating classical presynaptic inhibition onto Ia afferents.

Evidence for CB_1_Rs within the motor output apparatus becomes more direct in the ventral horn and at the neuromuscular junction. A primate study reported CB_1_R labelling in ventral horn, specifically on the somatodendritic part of motoneurons [53]. Notably, this finding diverges from the predominant localization of CB_1_Rs on axon terminals where they modulate presynaptic membranes [15,54]. Thus, even though ultrastructural work in primate spinal cord reports CB_1_R immunoreactivity in dendritic profiles, these postsynaptic CB_1_Rs on α-motoneurons are still likely to represent only a smaller fraction of the overall CB_1_R pool within ventral-horn circuitry. Nonetheless, cannabinoids do influence the presynaptic side of motor output by acting directly at motor terminals on peripheral neuromuscular junctions. In vertebrate NMJ preparations, such as a lizard and frog NMJ models, endocannabinoids reduced presynaptic calcium transients and transmitter release in a manner consistent with presynaptic CB_1_R-mediated control of neurotransmission [55,56]. On the postsynaptic side of the junction, CB_1_Rs do not seem to be present, even if some CB_1_Rs have been reported in skeletal muscle at subcellular sites such as the mitochondria [57].

Primary localization studies reported little or no CB_2_R immunoreactivity in both the DRG or spinal cord. CB_2_Rs become detectable only after nerve injury or stress, which includes some CB_2_R induction in DRG and its ipsilateral dorsal horn [58]. CB_2_R upregulation, however, is typically not linked to neurons but to activated microglia and astrocytes [59,60]. Accordingly, basal CB_2_Rs in spinal reflex circuitry and tract-terminal CB_2_Rs in descending pathways remain unsupported.

### 4.4. Involvement of CB Receptors in Supraspinal Modulatory Tracts

Besides segmental spinal circuitry, cannabinoid receptors might also influence descending spinal pathways modulating the excitability of this circuitry. CB_1_R expression is demonstrable in multiple brainstem nuclei. It is present in the vestibular nuclei [61,62], which give rise to vestibulospinal pathways, and in the pontomedullary reticular formation [63], which forms the source of reticulospinal pathways. Additionally, CB_1_Rs are present in monoaminergic nuclei, including the raphe nuclei [64] and the locus coeruleus [65], whose monoaminergic projections modulate segmental spinal circuitry. Nevertheless, even though the signal is present, there is currently no evidence that CB_1_Rs are located on somatodendritic parts of the actual descending neurons modulating spinal reflex circuitry and not just on axon terminals from other sources terminating in those nuclei. Furthermore, CB_1_Rs neither seem to be expressed on the axon terminals of the descending tracts located in the spinal cord. CB_1_R immunoreactivity is prominent in the dorsolateral funiculus and in dorsal horn neuropil. However, interrupting descending input by a rostral spinal cord hemisection in rats only produces a minor change in CB_1_R immunoreactivity, thus arguing that most CB_1_R-mediated signals do not come from severed descending systems [52]. The influence of CB_1_Rs on the supraspinal modulation of segmental spinal circuits is therefore, at best, mediated only indirectly.

## 5. Applying the Distribution of Cannabinoid Receptors to Motor Disorders

Cannabinoid receptors are positioned at strategic control nodes within motor circuitry. As a result, the modulation of cannabinoid signaling could, in principle, influence the major domains of motor function, spanning the selection and execution of movement as well as the regulation of skeletal muscle tone. This positioning suggests that cannabinoids may offer symptomatic benefit across different motor manifestations. Accordingly, they have been investigated clinically across mechanistically diverse conditions, most commonly in movement disorders and among the tone disorders, particularly in those resulting in spasticity. On the basis of receptor distribution along motor pathways, together with receptor-type-specific signaling properties, it is possible to infer how cannabinoid signaling may influence the final motor output. Together with a neuroanatomical understanding of clinical signs in motor disorders (see Box 2), one could implicate what disorders are expected to respond to treatment with cannabinoids. A summary comparing the proposed effects of cannabinoid signaling on motor outcomes with clinical outcomes is presented in Table 1. Based on their anatomical distribution, both CB_1_R and CB_2_R may be involved in motor control. Although CB_2_R expression is reported within elements of motor circuitry, effects on motor manifestations are more readily inferred from CB_1_R distribution. CB_1_Rs show markedly higher and more consistently observed expression in relevant neuronal compartments. In contrast, CB_2_Rs are generally better conceptualized as neuroprotective modulators. Any impact on motor disorders is more likely to arise indirectly through anti-inflammatory and survival-promoting actions, rather than directly affecting their motor signs [11].

Box 2 **Neuroanatomical basis of motor disorders.**

**Neuroanatomical basis of motor disorders**
 Motor disorders reflect a dysfunction anywhere along the motor pathway, from the motor cortex and basal ganglia through the brainstem and spinal cord to peripheral nerves, neuromuscular junction, and muscle. When the pathology affects the basal ganglia, for instance, in Parkinson’s disease and Huntington’s disease, the result is the broad family of classic movement disorders, including Parkinsonian syndromes, choreas, dystonias, and dyskinesias (see Box 1). Lesions involving the spinal cord reflex circuitry primarily affect muscle tone. For instance, lesions of upper motor neurons give rise to spastic weakness, as inhibitory supraspinal modulation of spinal reflexes is compromised. Such lesions may follow stroke, upper motor neuron disease, or spinal cord damage. When the pathology shifts to the lower motor neurons, as in spinal muscular atrophy, the clinical picture changes to flaccidity, as both spinal reflex arches and voluntary movement are unable to reach the skeletal muscle. Disorders rooted in the cerebellum primarily manifest coordination impairment and are seen as ataxic syndromes (see Box 1). The anatomically most peripheral group of motor disorders consists of neuromuscular junction disorders, in which impaired synaptic transmission leads to fatigable weakness and primary disorders of the muscle itself, collectively termed myopathies. Although typically considered as motor disorders, myopathies are primarily cell-intrinsic disorders of the effector organ, whereas others are predominantly disorders of neuronal circuits that control movement.

According to the proposed CB_1_R localization in basal ganglia, CB_1_R signaling is expected to suppress thalamocortical drive and reduce abnormal movement, despite some pro-kinetic actions on the indirect pathway. On corticostriatal terminals, CB_1_R agonists reduce glutamate release onto D_1_-MSNs, so these neurons are less effectively recruited by cortical motor commands. On D_1_-MSN axon terminals in GPi and SNr, CB_1_R activation suppresses GABA release, relieving the inhibition of GPi/SNr neurons and thereby strengthening their inhibitory output to the thalamus. A smaller CB_1_R population on local D_1_-MSN axon collaterals in the striatum reduces GABAergic self- and lateral inhibition, which favors D_1_-MSN firing. However, this effect is probably minor compared with the strong presynaptic control at the corticostriatal synapses. Because nigrostriatal dopaminergic neurons lack CB_1_Rs, dopamine itself is not directly modulated.

Inferring from this CB_1_R distribution in basal ganglia, CB_1_R agonists are expected to modulate and ameliorate abnormal hyperkinetic movements, clinically seen as tics and other dyskinesias. Hypokinetic movements, on the other hand, are not expected to be affected from the proposed distribution (see Box 1). Interestingly, within movement disorders, systematic reviews show that the most relevant clinical benefit of cannabinoids is observed in tic disorders such as Tourette syndrome, where cannabinoids may reduce tic severity and the premonitory urge in some patients [66,67]. For Parkinson’s disease, which is primarily a hypokinetic movement disorder, clinical results have generally been mixed or negative. When improvements occur, they tend to be modest, variable between individuals, and not reliably reflected in objective motor scales [68,69,70]. Such variability arises from levodopa-induced dyskinesias, which are a hyperkinetic treatment complication and are in theory expected to respond to cannabinoid treatment. A clinical study using a synthetic THC derivative, nabilone, actually reported the reduction in such dyskinesias [71], whereas a controlled crossover study using an oral cannabis extract found no improvement [72]. Furthermore, movement disorders like Huntington’s disease, although primarily hyperkinetic, also show mixed clinical results, despite CB_1_R distribution alone possibly suggesting a potential benefit [68,73,74]. These findings highlight two points. First, hyperkinesia does not automatically predict a reproducible response to cannabinoids. Second, different cannabinoids may affect motor manifestations in different ways. In clinical studies, different cannabinoids are used, most commonly THC, CBD, nabiximols, and nabilone, which may differ in efficacy for specific clinical manifestations. If a compound acts mainly through CB_1_R, such as nabilone, CB_1_R localization may provide a reasonable first approximation of the expected motor modulation. In contrast, oral cannabinoids, CBD especially, have lower affinity for CB_1_R, so CB_1_R distribution alone is unlikely to predict its clinical impact. More broadly, even with the receptor profile considered, alignment with the observed clinical outcomes remains difficult. Most movement disorders combine hyper- and hypokinetic elements. Symptomatic therapies, especially dopaminergic treatments, further reshape motor output. As a result, clinical data rarely allow for a clear inference about how cannabinoids modulate hyperkinetic versus hypokinetic manifestations in isolation.

In the case of muscle tone, cannabinoids are primarily expected to lower it, according to the proposed receptor localization in spinal circuits. By activating presynaptic CB_1_R on axon terminals within spinal circuitry, cannabinoids reduce transmitter release onto spinal networks. This way, both the motor output to extrafusal muscle via α-motoneurons and likely also γ-motoneuron-related gain control of spindle sensitivity are dampened. A smaller additional effect may come from postsynaptic CB_1_R on α-motoneuron dendrites, which could modestly reduce motoneuron excitability. Peripherally, presynaptic CB_1_R at the neuromuscular junction can reduce acetylcholine release, further lowering effective muscle activation. CB_1_R within spinal interneuron networks can shift motoneuron drive in either direction: suppressing inhibitory interneuron output may increase reflex gain, whereas inhibitory–inhibitory wiring can strengthen downstream inhibition and reduce gain. Consequently, its net effect on tone is difficult to infer from localization alone and may be secondary to other presynaptic CB_1_R sites.

**Table 1:** Observed clinical outcomes vs. the proposed effects of cannabinoids on motor manifestations.

Motor Disorders		Clinical Outcomes	Refs.	Proposed Motor Outcomes *
Movement	Hyperkinetic disorders	Beneficial effects observed, especially in tics in Tourette syndrome	[66,67,74]	Reduced movement
	Hypokinetic disorders	Mixed or negative results	[68,69,70,74]	
Tone	Increased tone	Beneficial effects observed, especially in spasticity related with multiple sclerosis	[75,76,77]	Lowered muscle tone
	Decreased tone	Not enough clinical data		

* Proposed outcomes are based on CB_1_R distribution in basal ganglia (see Figure 1) and spinal cord circuits (see Figure 2). Background colors are used to more readily distinguish subtypes within each motor disorder group.

Inferring from CB_1_R distribution in spinal circuits, CB_1_R agonists are therefore expected to ameliorate symptoms of increased muscle tone, clinically observed as spasticity. Consequently, it is partially to be expected that clinical studies are showing reproducible therapeutic effects of cannabinoids in spasticity syndromes (see Box 1). The best and most reproducible clinical effects of cannabinoids are observed particularly in multiple sclerosis [75,76] and to some lesser extent in motor neuron disease-related spasticity [78]. In these settings, cannabinoids consistently improve patient-reported stiffness, frequency, and intensity of involuntary spasms. Accordingly, in several jurisdictions, cannabinoid-based medicines, nabiximols, are approved for the symptomatic treatment of spasticity in multiple sclerosis [79]. Nevertheless, due to the same limitations as those discussed in movement outcomes, we cannot expect the proposed distribution-based predictions to align consistently with all tone abnormalities. At least for post-stroke, spinal cord injury, and cerebral palsy-associated spasticity, the evidence of cannabinoids’ efficacy is currently smaller and more heterogeneous [5,74,77,80].

In both the basal ganglia and spinal circuits, available evidence suggests that CB_2_R agonists play a comparatively minor role, consistent with lower receptor expression density. According to their distribution, they are expected to exert weaker but more selective circuit effects, most notably by reducing SNc dopamine neuron firing and STN glutamate output. Nevertheless, CB_2_Rs are still best understood as neuroprotective modulators whose long-term impact on motor function in basal-ganglia disease stems more from anti-inflammatory and survival-promoting actions than from acute, global changes in motor circuit excitability [11]. CB_2_R should therefore be discussed primarily in the context of disease modification, rather than the symptomatic treatment of motor manifestations.

Finally, several important limitations should be acknowledged when inferring motor outcomes from receptor localization. Despite inclusion of the major motor circuits involved in motor control, the predictive model remains a simplification. Basal ganglia function emerges from an interacting network, meaning that the same agonist may act at several sites with opposing consequences, making the overall motor effect difficult to predict. This problem is also further compounded by the fact that cannabinoids act outside the basal ganglia and spinal circuits including in the cortical, cerebellar, and brainstem circuits. Clinical effects therefore reflect broader actions such as sedation and altered coordination, both affecting motor manifestations in clinical practice. Disease itself also modifies receptor expression, coupling, and circuit balance, so static receptor maps are a better predictor of clinical outcomes in physiological conditions. It is also worth noting that much of the mechanistic rationale relies on rodent CB_1_R mapping and physiology. Cannabinoid receptor expression and cellular localization differ between species, thus limiting direct translation to humans [81].

## 6. Conclusions

Because cannabinoid receptors occupy strategic control points within motor control circuitry, cannabinoids can modulate a broad spectrum of motor manifestations, ranging from effects on movement execution to effects on skeletal muscle tone. Given the distribution of cannabinoid receptors within basal ganglia circuits, together with their inhibitory signaling properties, cannabinoids are expected to bias motor output toward reduced movement. Their localization within spinal motor reflex circuits, in turn, suggests a capacity to reduce muscle tone. By understanding the characteristic motor manifestations of individual diseases, one can infer the potential therapeutic utility of cannabinoids for the symptomatic treatment of motor disorders. Overall, the proposed distribution-based expectations align with clinical observations. Cannabinoids appear most plausible for hyperkinetic disorders, whereas effects on hypokinetic signs, such as those seen in Parkinson’s disease, tend to be less consistent. With respect to muscle tone, cannabinoids would likewise be expected to improve spasticity, consistent with their clinical use in multiple sclerosis. In this context, CB_1_Rs likely exert a stronger influence on motor function, as they are more abundantly expressed within motor circuits compared to CB_2_Rs and can therefore better modulate circuit activity.

For a more comprehensive prediction of the effects on motor control, cannabinoid receptor distribution in the cerebellum should also be examined. This could also extend distribution-based inferences to ataxic disorders. In addition to rodent studies, further work in non-rodent species, ideally including primates and human tissue, would help define cannabinoid receptor distribution more precisely. Based on the proposed findings on receptor distribution, we suggest that future studies evaluate potent CB_1_R agonists for the symptomatic treatment of motor manifestations. For CB_2_Rs, studies using selective CB_2_R agonists are needed to better define their contribution to motor function and avoid CB_1_R-mediated effects overshadowing the observed outcomes. As selective CB_2_R agonists are not yet available for human use, initial studies should be conducted in animal models.

## Data Availability

No new data were created or analyzed in this study. Data sharing is not applicable to this article.

## Abbreviations

The following abbreviations are used in this manuscript:

CB_1_R	Cannabinoid receptor 1
CB_2_R	Cannabinoid receptor 2
2-AG	2-arachidonoylglycerol
AEA	Anandamide
CNS	Central nervous system
GABA	Gamma-aminobutyric acid
MSN	Medium spiny neurons
GPi	Globus pallidus internus
SNr	Substantia nigra pars reticulata
LMN	Lower motor neuron
GPCR	G protein-coupled receptor
D_1_-MSNs	D_1_ receptor-expressing medium spiny neuron
D_2_-MSNs	D_2_ receptor-expressing medium spiny neuron
STN	Subthalamic nucleus
SNc	Substantia nigra pars compacta
5-HT_2_	5-hydroxytryptamine
EPSC	Excitatory postsynaptic current
NMJ	Neuromuscular junction
DRG	Dorsal root ganglion
